# The Pathology and Diagnostic Value of Pain

**Published:** 1898-04

**Authors:** J. Clarence Johnson

**Affiliations:** Atlanta, Ga.


ATLANTA
Medical and Surgical Journal.

Vol. XV. APRIL, 1898. No. 2.

L. B. GRANDY, M.D., } EDITORS.

DUNBAR ROY, M.D., }

M. B. HUTCHINS, M.D.,
BUSINESS MANAGER.

### ORIGINAL COMMUNICATIONS.

### THE PATHOLOGY AND DIAGNOSTIC VALUE OF PAIN.

By J. CLARENCE JOHNSON, M.D.,
Atlanta, Ga.

In the crowding cares of a doctor's life he has little time for study of many questions that vitally concern the exactness of his work. We are therefore inclined to accept the relics of our predecessor's faith, and follow beaten paths that lead to oft-repeated disappointments. Made restless by defeat, we turn with exaggerated zeal to new remedies and methods that promise improved results, and with devotion thus misplaced, neglect the material elements of scientific progress and success.

The topic which I have chosen for discussion may seem out of date; but faces never grow so familiar that some feature may not be lost to memory or dimmed beyond the probability of prompt and perfect recognition. So, there is no principle in medicine so well established that it may not be controverted, no fact that may not be modified by eager search for truth. It is not amiss to take an occasional inventory of our knowledge, and circumspectly watch our way, though apparently not in need of thought or care.

There are many physicians who have never seen a case of cholera, but there is not one who fails to witness suffering every day. It is such a common attendant of disease that we are apt to regard it an essential part, worthy only of that consideration which the patient has a right to expect or demands towards its relief.

The highest office of a physician is embodied in the well-known maxim, "*Curare cito, tuto et jucunde.*" Especially is it our duty to accomplish the last when consistent with a safe and rapid cure. But the ready means at our command are too often abused in our haste to respond to the solicitude of sympathizing friends, or the urgency of the sufferer himself, thereby obscuring an important factor in accurate diagnosis.

Pain is the natural language of an organ diseased, as is the cry of a babe its speech for hunger, or indication of distress; and its character, like the character of the cry, points to the seat and nature of the cause.

You are all too familiar with the various forms of pain for me to dwell upon them here. It is enough to say that they differ with the structure of the part diseased, its relation to other parts, and the degree and extent of morbid action which produces them. Unfortunately, the description of pain must be vague at best, because it cannot be seen, nor can its intensity be measured or expressed. It can only be felt and estimated by the patient himself. To him exactness is impossible, for it so engages his power of resistance that the departure from the normal state of conscious sensation is magnified. While its manner is obvious, and more or less consistent, it is influenced by the habit of the subject and mental agitation. So that, pain of the same degree and character, arising from a similar cause and condition, appears more violent in one individual than another, or seems to exist where it is not present, or an actual part of the disease.

Yet, when brought to the last analysis, we must admit that so-called imaginary ills are practically real, though the nature of their reality may escape our detection or baffle our comprehension. We cannot state positively that a patient has or has not pain. We can only know by scientific reason that it is a natural sequence or attendant of certain morbid actions, and apply the evidence adduced to the determination of its presence, severity, and character.

Hence the ignorance that veils the pathology of diseases in which pain is the predominant or exclusive symptom—bearing a name that only indicates its seat and existence.

Pain is not an entity, not a disease, but a part and product of a pathological process, the physiology of abnormal sensation, the symbol of perverted function; as delirium is of disordered intellection, and convulsions of incoördinate muscular contraction.

Therefore, in seeking to ascertain the cause of pain and the manner of its production, we must look to the source and conduct of normal sensation. It is thus rendered easily apparent that it arises from perverse generation or distribution of sensory impressions, in any event involving a material change in the part or parts affected.

Engorgement of the meninges may excite central or peripheral pain, but preceding or attending the engorgement is an alteration of metabolic, cardiac, or vascular action.

Normal sensation consists in the reception by a sensory ganglion, or cell, of an impression made upon a tactile, Pacinian, or Veta's corpuscle, or an end bulb, conveyed thither by a centripetal filament. It becomes a conscious sensation when it reaches the cerebrum. While reflex action from peripheral irritation may give rise to the sensation of pain at the point of irritation, there is in reality no pain without intelligent reception—as there is no sound from concussion of the air without an ear to hear it.

The relation, then, which any part or organ of the body sustains to other parts is rendered conscious and appreciable by the exercise of the sensory functions, as exhibited in the phenomena of health and disease. Anything that increases the impressionability of the sensory corpuscles or receptivity of the sensory ganglia, lessens to the same extent the general power of resistance and favors the production of pain.

The normal action of the sensory corpuscles or ganglion, like the function of any organ, depends upon a normal state of nutrition, which in turn depends upon the quantity and quality of its blood supply, and the elective and appropriative capacity of its constituent cells. So, their susceptibility may be increased or diminished by a perverted nutrition—the result of direct interference with their metabolism, as from violence or pressure, or a vitiated pabulum.

Direct irritation of a sensory ganglion does not cause pain. A ganglion can only receive the impression, and by reference to other parts, through an efferent filament, transform it into a sensation. The ultimate effect of the impression is governed by the manner of reception and transmission to a conscious center, and the tolerance of the general system, the power of resistance which is or can be exercised. The nature of the tolerance and extent of resistance is inversely proportionate to the susceptibility of the individual; the more highly organized, being more susceptible, have less, and *vice versa*. By the same associative action are the reflex manifestations controlled.

If, then, from any cause, the function of a corpuscle or ganglion is exalted, the intensity of the sensation will be exaggerated, as emotion inflames a mind diseased, or light affects an irritated eye. The converse is true. If the function is restrained, the intensity of the sensation will be lessened, as is demonstrated by the action of anesthetics and anodynes.

So, in organic changes thus affecting the sensory surface and centers, impressions that would create no pain or awaken no conscious sense may induce severe discomfort. The habit of conduction, the relation of the sensory, motor, and trophic systems is emphasized, and, in consequence, the functional activity of the parts which they in common supply is deranged.

It thus appears, that in the pathology of pain not only the corpuscles and the ganglia, but the whole economy, is involved; that each organ contributes to the general power of resistance, and in so doing surrenders a proportionate degree of its vital capacity and official exercise. The expression of pain is the result of this concerted action.

By applying these observations to the various forms of pain, and the conditions from which they arise, we will be able to estimate their diagnostic value and importance.

For convenience, pain may be divided into three classes:

1. That occurring in the absence of appreciable organic change in the tissues involved, being the chief or exclusive symptom.

2. That occurring without appreciable organic change, attended by other symptoms.

3. That accompanied by organic change, and obviously dependent upon it.

We are all familiar with the nature of pain as it occurs in the process of inflammation, and know that it is the result of irritation of the end filaments of the nerves by the products of the morbid action, in which the filaments themselves participate. We know that the character of the pain is governed by the abundance of the nerves and the presence or absence of elastic tissue in the part diseased. Thus we are enabled to distinguish the pains of pleurisy and pneumonia, of cellulitis and whitlow.

Our task is not so easy when we attempt to comprehend the meaning and explain the value of pain in the absence of organic change.

A man eats heartily; soon thereafter he is seized with gastric pain, or pain between the shoulder blades, or in the dorsal region of the spine. We know that indigestion is the cause, but how? There was no previous engorgement of the capillaries beyond the normal. There is no excess or lack of acid sufficient to so irritate the sentient surface. It is not produced by mechanical pressure of the food, because at another time he eats more heartily without the least discomfort. Besides, if this were so, pain would follow normal distension of the bladder, or ordinary active thought. The explanation rests in the fact that the whole anatomy of the stomach is engaged in the effort to discharge its function, each constituent part contributing more than its usual share, each thus becoming more sensible of impressions and disposed to perversion. The nerves, with susceptibilities increased, exaggerate the impression they receive and direct them to their centers, whence abnormal force is discharged to the muscular coats, and spasm is the result. Irritation is intensified. The impressions are conveyed to the spinal ganglia by the splanchnic nerves, and here are formed sensations which are reflected to the back and stomach, recognized as aches and pains.

The same process substantially obtains in astigmatic pains, and in myalgia from muscular exhaustion, and in neuralgia from any cause that similarly affects the activity or integrity of the nerve supply, as anemia or malaria. It likewise follows inordinate mental exercise, worry, or overwork.

But it is that form which attends febrile and other diseases of idiopathic nature that is most difficult of comprehension.

Fever affects every part and function of the body. Pain is an initiatory symptom. Does the elevated temperature cause the pain, or is the pain associated in the production of the fever? The solution of this question will throw important light upon the expediency of agents which at the same time reduce the fever and allay the pain, as the coal-tar preparations.

There are remedies that will relieve the pain and not reduce the fever. There are also those which will reduce the fever and not remove the pain. Besides, the intensity of the pain is not always measured by the degree of fever. It is true that the centers which control the generation of heat and preside over common sensations are intimately related, so that one can hardly be affected without disorder of the other. Yet it is in the general departure from the healthy state alone that both can have their source, and in the operation of the exciting cause must we find the answer to our question.

In the action and reaction of the febrile process, the sensory, motor, and trophic systems are all involved. Compensatory functions are engaged to supply the needs ot organic life. Voluntary action is economized. Every power is enlisted to thwart the insidious decay. Yet waste exceeds repair. Vitality is lowered, conduction of impressions is opposed, sensation is obtunded, and pain is lost to consciousness, or disappears. But the fever rises higher, and continues until nature is subdued or reasserts her sway.

It is not within the province of this paper to consider at length special pains or pains attending special diseases. A few examples will suffice to illustrate the principles I have undertaken to establish.

An arm is amputated. Months or years subsequent to the operation pain is felt in the part removed. The spinal meninges are inflamed, the patient suffers pain throughout the thorax, abdomen, and extremities at points and in directions of the nerves which are impressed.

Disease of the hip-joint causes pain in the knee, and dislocation of the humerus pain in the hand, demonstrating the fact that sensation is referred to the extremity of a nerve. But pain is not always thus disposed. According to its intensity it is local, symmetrical or generalized; and a knowledge of its source and meaning only requires careful tracing of the filaments—through which it is reflected—to their ganglionic centers, and the distribution of other filaments having the most intimate anatomical and physiological relation to them.

Pain in the ear is a common symptom of a carious tooth, which is easily explained by the fact that both are supplied with sensation by the trigeminal nerve. So it is with neuralgia of the eye and other parts of the face.

The frequency of pain in the head is due to constant exercise of the functions by which directive agency is maintained over other organs of the body. The causes are manifold, but the pathology is essentially the same.

One of the most interesting forms of pain is that attending labor. Labor is ordinarily a physiological process. The pain is consonant with uterine contraction, and is supposed to be caused by pressure on the nerves. Be that as it may, it is obvious that there are other factors in its production. First, an impression must be made upon the sensory supply, which being conveyed to the sacral ganglia, originates an impulse that is sent to the muscular fibers of the womb, and they contract. New impressions thus arise, with similar results, and these successive acts, favored by the susceptibility of the pregnant state, and the general contribution to the expulsive efforts, intensify the pain. Whether we should class this as pathological will depend upon our construction of the divine injunction, "In sorrow shalt thou bring forth children."

Finally, what is the pathology of hysterical pain? We have a patient who complains that she has pains of this or that kind, here or there. There is no acceleration of the pulse, perhaps, no rise of temperature, no objective symptoms that confirm the truth of her assertion. We have no doubt that she is suffering, or believes it strongly. We have no doubt there is a cause for this effect, and we term it diseased imagination. But has she really pain?

There are other symptoms of hysteria that will aid in our decision. No one doubts a phantom tumor, or the occurrence of eclampsia. We all know that these phenomena are possible only by diseased fasciculi, abnormal generation or distribution of motor force. What is delirium tremens but disordered intellection and reception of diseased impressions—the natural sequence of metabolic inhibition or perversion. Truly does the drunkard's eye, in fiery frenzy rolling, body forth the forms of things unseen, and give to airy nothing a menacing presence and a name. But to his tortured brain the snakes and devils are as real as if he felt their poisonous fangs, or stifled in their ruthless clutch.

Pain is consciousness diseased, or knowledge of diseased sensation. The mind cannot form impressions; it can create thought and govern their relation. All ideas in a healthful mental state are consistent with the natural sense. Therefore, if pain apparently exists, it must have reality and a cause somewhere.

So is pain produced. So are its secret ways made manifest, and by its character and conduct is its value known. Though intangible and unseen, it betrays its habitation and source of power, and enables us to offer open combat to man's most potent and relentless foe.

